# The effect of the ragweed sublingual immunotherapy tablet MK-3641 on rescue medication use

**DOI:** 10.1186/1710-1492-10-S2-A32

**Published:** 2014-12-18

**Authors:** Sandra Gawchik, Peter Creticos, Kevin R Murphy, Gary Berman, David I Bernstein, Jennifer Maloney, Amarjot Kaur, Hendrik Nolte

**Affiliations:** 1 Asthma & Allergy Associates, Chester, PA, USA; 2 Department of Medicine, Johns Hopkins University School of Medicine, Baltimore, MD, USA; 3 Boys Town National Research Hospital, Boys Town, NE, USA; 4 Minneapolis Allergy & Asthma Specialists, Minneapolis, MN, USA; 5 Bernstein Allergy Group, Cincinnati, OH, USA; 6 Merck & Co., Inc., Whitehouse Station, NJ, USA

## Background

Allergic rhinitis with/without conjunctivitis (AR/C) sufferers often rely on pharmacotherapy to relieve symptoms. Although the main goal of immunotherapy is long-term disease modification, reducing or eliminating the need for pharmacotherapy is also an important and desirable treatment goal.

## Methods

Data were pooled from two trials that evaluated the efficacy and safety of short-ragweed sublingual immunotherapy tablet (SLIT-T), MK-3641 (*Ambrosia artemisiifolia*; Merck/ALK-Abelló). Subjects with ragweed-pollen–induced AR/C were randomized ~16 weeks before the 2010 pollen season to once-daily MK-3641 (6 or 12 Amb a 1-U doses; one trial also included a no-effect dose of 1.5 Amb a 1-U) or placebo. During the trial, all subjects, whether taking MK-3641 or placebo, could use AR/C rescue medication, including oral/ocular antihistamines and intranasal/oral corticosteroids. We examined rescue medication use in all groups.

## Results

In pooled results from the two studies, 159 of 318 (50.0%) subjects receiving MK-3641 12 Amb a 1-U and 144 of 324 (44.4%) subjects receiving 6 Amb a 1-U used no rescue medication over the entire ragweed season, compared with 109 of 340 (32.1%) subjects receiving placebo. These differences represented 56% and 38% improvements over placebo. Similarly, during the peak ragweed season 173 of 311 (55.6%) subjects and 161 of 317 (50.8%) subjects in the 12 Amb a 1-U and 6 Amb 1-U groups, respectively, reported no rescue medication use, in contrast to 136 of 333 (40.8%) subjects receiving placebo. Fewer subjects taking 12 and 6 Amb a 1-U (28% and 19%, respectively) used oral antihistamine than those taking placebo; 35% and 28% fewer subjects used ocular antihistamine; and 43% and 27% fewer subjects used intranasal corticosteroid (oral corticosteroid was used by <5 subjects in any group, so rates were not calculated).

## Conclusions

Compared with placebo, the SLIT-T treatment MK-3641 reduced rescue-medication use among subjects with ragweed-pollen–induced AR/C.

## Trial registration

ClinicalTrials.gov Identifiers: NCT00783198; NCT00770315

